# Inoculation of Animals for the Prevention of Pleuro-Pneumonia

**Published:** 1883-04

**Authors:** 


### Art. XVII.—INOCULATION OF ANIMALS OF THE BOVINE SPECIES FOR THE PREVENTION OF CONTAGIOUS PLEURO-PNEUMONIA BY INJECTION INTO THE VEINS.

*Condensed from the Veterinarian, February,* 1883.

BY PROFESSORS THIERNESS AND DEGINE.

If there exists a scientific movement which deserves to be noticed and approved it is assuredly that which we see at this present time becoming known to the learned men of different countries in order to arrive at the solution of various questions relating to the nature and proplylectic treatment of contagious diseases.

In this general movement we especially remark the experimental investigations which are connected with inoculation as a preventive of these diseases.

The efficacy of inoculation for a long time has been an established fact for small-pox in the humans as well as for sheep. Recent well-known investigations have established the fact that chicken cholera, anthrax and symptomatic anthrax in domestic animals can be prevented, or modified so as to be harmless.

Similar results are said to be obtained by the inoculation of cattle for the prevention of pleuro-pneumonia. In a scientific point of view this question is virtually established, but it cannot be said to be so from a practical point of view, for as yet the fact is not well established that its application is really beneficial, that is to say economical.

In the first place the operation is not without danger to the animal operated upon. Again, its preventive action is neither constant nor certain.

Inoculation, so far as performed by us, consists simply of the intravenous injection of the virulent liquid obtained from the lungs of pleuro-pneumonic subjects. It should be taken *at the time of the inoculation* from some diseased portion of the lung which is entirely free from septic matter.

The best results are obtained by cutting the pulmonary tissue in several directions, and then pressing out the virulent serum with the hand.

The liquid thus obtained is somewhat cloudy and sanguineous and should be subsequently filtered through fine linen, after which it should be drawn up into a Pravaz syringe two grammes size.

The animal to be inoculated should be thrown in a convenient position on the straw. The hair should be cut off round the outside of the jugular vein near the throat on one side or the other.

An assistant should place his hand on the lower part of the external jugular vein so as to distend it.. This being done the skin is pinched up in order to make an incision. That will enable us to more readily detach the adjacent cellular tissue, and uncover completely the before mentioned vessel. The needle of the already well charged syringe should now be introduced transversely into the wall of the extended vein and its contents slowly injected. As soon as completed the pressure should be removed from the vein. A twisted suture made with two pins completes the operation.

